# A Simple and Effective Method for Construction of *Escherichia coli* Strains Proficient for Genome Engineering

**DOI:** 10.1371/journal.pone.0094266

**Published:** 2014-04-18

**Authors:** Young Shin Ryu, Rajesh Kumar Biswas, Kwangsu Shin, Vinuselvi Parisutham, Suk Min Kim, Sung Kuk Lee

**Affiliations:** 1 School of Energy and Chemical Engineering, Ulsan National Institute of Science and Technology (UNIST), Ulsan, Republic of Korea; 2 School of Biological Sciences, Ulsan National Institute of Science and Technology (UNIST), Ulsan, Republic of Korea; Charité-University Medicine Berlin, Germany

## Abstract

Multiplex genome engineering is a standalone recombineering tool for large-scale programming and accelerated evolution of cells. However, this advanced genome engineering technique has been limited to use in selected bacterial strains. We developed a simple and effective strain-independent method for effective genome engineering in *Escherichia coli*. The method involves introducing a suicide plasmid carrying the λ Red recombination system into the *mutS* gene. The suicide plasmid can be excised from the chromosome via selection in the absence of antibiotics, thus allowing transient inactivation of the mismatch repair system during genome engineering. In addition, we developed another suicide plasmid that enables integration of large DNA fragments into the *lacZ* genomic locus. These features enable this system to be applied in the exploitation of the benefits of genome engineering in synthetic biology, as well as the metabolic engineering of different strains of *E. coli*.

## Introduction

Recombination-mediated genome engineering tools have proven their utility in synthetic biology and metabolic engineering [1]–[3]. Simultaneous editing of multiple loci on the chromosome using short single-stranded oligo nucleotides (nt), called multiplex automated genomic engineering (MAGE), has recently become a unique genomic engineering tool that can aid in efficient and accelerated evolution of desired functions over a short time periods [4]. Such genome engineering tools could facilitate the understanding of certain basic biological phenomena and be used to generate highly functionalized organisms with well-defined genotypes that cannot be easily generated by natural evolution [5]. The method utilizes the λ Red recombination proteins (Gam, Exo, and Beta) and oligo nucleotides to introduce desired mutations on the bacterial genome [6]. In addition to the recombination system, inactivation of the methyl-directed mismatch repair (MMR) system is generally necessary to avoid recognition and correction of desired mutations by the native MMR system.

Genome engineering approaches have been vastly improved with the rapid development of purpose-specific strains. Using MAGE, all 314 of the TAG stop codons in *Escherichia coli* were replaced with TAA stop codons, thus creating a codon space for use with non-natural amino acids [7]. A 20% improvement in indigo production has been achieved with the simultaneous introduction of a T7 promoter into 12 genetic loci [8]. In the largest example of MAGE cycling (110 cycles), the His-tag sequence has been inserted into 38 essential genes encoding the entire translational machinery of *E. coli*, thus facilitating the *in vitro* reconstruction of a translational machinery with high purity [9].

Although several modified *E. coli* strains (EcNR2, DY330, and EcHW24) are available for genome engineering purposes [10]–[12], these strains possess several disadvantages including disordered cell growth owing to the cytotoxic genes of defective prophage (like *kill*) [13], [14] and a permanently inactivated MMR system that results in significant accumulation of undesired background mutations [2], [3], [15], [16]. In addition, the defective prophage is not easily portable to different *E. coli* platforms, considerably hindering the full exploitation of MAGE functionality for extensive genome editing. Therefore, limitations exist in the application of MAGE to various custom-made *E. coli* strains. Moreover, the MMR system should be rescued in order to avoid accumulation of random mutations following MAGE.

In order to improve the utility of MAGE and its portability to different *E. coli* strains, we developed pRED suicide plasmids that possess all necessary components for oligo-mediated recombination and allow transient inactivation of the host MMR system via insertional inactivation of *mutS*. In addition, pINZ plasmids also provide options for introducing large heterologous genes into the chromosome. Thus, our system can aid in extending the power of MAGE for rewiring central metabolic processes or in modifying large heterologous genes.

## Materials and Methods

### Bacterial Strains, Culture Media, and Growth Conditions

The strains used are listed in Table 1, and *E. coli* MG1655 was used as the parental strain in this study. Strains were cultured in Luria Bertani broth (LB) at 30°C unless otherwise specified. For MAGE, *E. coli* strains were cultivated in reduced-salt LB media (5 g/L NaCl). Media were supplemented with suitable antibiotics at the respective concentrations (kanamycin [Km] at 50 µg/mL, chloramphenicol [Cm] at 30 µg/mL, or ampicillin [Amp] at 100 µg/mL). Cell growth was monitored by measuring the optical density at 600 nm (OD_600_) using a Libra S22 spectrophotometer (Biochrom Ltd., Cambridge, United Kingdom).

**Table 1:** *E. coli* strains and plasmids used in this study.

Strains/plasmids	Description/genotype	References/sources
**Strains**		
*E. coli* DH10B	F^−^ *mcrA* Δ(*mrr-hsdRMS mcrBC*) φ80d*lacZ*ΔM15 Δ*lacX74 deoR recA1 ara*Δ*139* Δ(*ara leu*)*7697 galU galK λ* ^−^ *rpsL endA1 nupG* Str^r^	Invitrogen
*E. coli* DB3.1*λpir*	F^−^ *gyrA462 endA1* Δ(*sr1-recA*) *mcrB mrr hsdS20* (r_B_^−^ m_B_^−^) *supE44 ara-14 galK2 lacY1 proA2 rpsL20* (Str^r^) *xyl-5* Δ*leu mtl-1* λ*pir* lysogen	[30]
*E. coli* MG1655	Wild-type	[21]
EcNR2	MG1655, *bio::bla*, lambda-Red1, *mutS*::Cm^r^	[5]
EcSIM1	genome integrated pRED-1 in MG1655	This study
EcSIM2	genome integrated pRED-2 in MG1655	This study
EcINZ1	genome integrated pINZ-1 in MG1655	This study
EcINZ2	genome integrated pINZ-2 in MG1655	This study
EcSIM1.1	EcSIM1 containing pINZ1-LYC04	This study
EcSIM1.1a–f	representative variants after 18 cycles of MAGE	This study
**Plasmids**		
pSIM5	pSC101-ts *ori*, Cm^r^, Red *gam*/*exo*/*beta*	[17]
pProbe[tagless]	pSC101-ts *ori*, Km^r^	[22]
pAC-LYC	pACYC184 containing *Erwinia herbicola crtEBI* genes	[23]
pAC-LYC04	pAC-LYC containing *Hematococcus pluvialis ipi* gene	[23]
pRED-1	pSIM5 carrying *mutS* fragment, pSC101-ts *ori*, Cm^r^	This study
pRED-2	pSIM5 carrying *mutS* fragment, R6Kγ *ori*,Cm^r^	This study
pINZ-1	pProbe[tagless] carrying *lacZ* fragment, pSC101-ts *ori*, Km^r^	This study
pINZ-1-LYC	pINZ-1 containing *Erwinia herbicola crtEBI* genes	This study
pINZ-1-LYC04	pINZ-1 containing *Erwinia herbicola crtEBI* genes and *Hematococcus pluvialis ipi* gene	This study
pINZ-2	pProbe[tagless] carrying *lacZ* fragment, R6Kγ *ori*, Km^r^	This study

### Plasmid Construction

Plasmids and primers that we used are listed in Tables 1 and 2, respectively. The suicide plasmid, pRED-1, was constructed by a simple modification of the pSIM5 plasmid [17]. A partial sequence (542 base pairs [bp]) of the *mutS* gene was PCR-amplified using forward (mutSF) and reverse (mutSR) primers containing a *Bgl*II site and the PCR products were subsequently inserted into *Bgl*II-digested pSIM5 vector. For pRED-2, the pSC101-ts origin of replication of pRED-1was replaced with the R6kγ origin of replication using the PCR-based SLIC (sequence and ligase independent cloning) method [18], [19]. Briefly, pRED-1 was amplified (except the pSC101-ts origin) using primers R6KF and RP4R, while the R6Kγ origin was amplified from a pKNOCK plasmid using the RP4F and R6KR primers [20]. The fragments were treated separately with T4 DNA polymerase, mixed at a 1∶2 ratio of vector to insert, and transformed into *E. coli* DB3.1 λ *pir* [21] to generate the pRED-2 plasmid.

**Table 2:** Primers used in this study.

Primers Name	Primer sequence
mutS F	GAGAGATCTAGCGAGCAATCATCGACACA
mutS R	TTCAGATCTAACTACCGGATGGCGACCTT
R6KF	ACGGCTGACATACTAGTGCCGCAAATCGCTGAATATTCCT
R6KR	GGCACTAGTATGTCAGCCGTTAAGTGTTCCTGT
RP4R	CCAAGCCAACCAGCATATGGGCAGATGAAAACGGTGTAAAAAAGAT
RP4F	GCCCATATGCTGGTTGGCTTGGTTTCATCAG
P1F2	ATGGATATCGTGTAGGCTGGAGCTGCTTC
P4R2	CACCGATCGCTGTCAAACATGAGAATTAATTCCGGACGTCCGTCGACCTCGAGTTCGAAGTTCC
lacZF1	GCAACTCGAGTTAACGCCGTGCGCTGTTCG
lacZR1	TCCGGACGTCTGGAAAAACTGCTGCTGGTG
lacZF	ATCAGATCTTGATCCCCTGCGCCATC
lacZF2	GATAGCCCAGTAGCTGACATTCATCTGGTTGGCTTGGTTTCATC
lacZR2	TTAACTCGAGTTATGTCAGCCGTTAAGTGTTC
lacZ-MAGE	GGAAACAGCTATGACCATGATTACGGATTCACTGGCCGTCGTTTGACAACGTCGTGACTGGGAAAACCCTGGCGTTACCCAACTTAATCG
Ptac-lacZY	CAATTTCACACAGGAGATATCATATGACCATGATTACGGATTCAC
P4R	CTGTCAAACATGAGAATTAA
pKD13R	ATGAATGTCAGCTACTGGGCTATC
Cm_R	CCGTTTTCACCATGGGCAAATATTATACG
mutS seqF	GCACTAATCCTGCGGAACTG
Trc F	GAGCTGAATTCGATCTGGTTTGACAGCTTATCATCGA
crtB R	GCAGTCGACCTAAACGGGACGCTGCCAAAGACC
crtE F	GAGCTGAATTCAATTCTCATGTTTGACAGCTTATCATC
dxs MAGE	GCGGACTACATCATCCAGCGTAATAAATAAACAATAAGTATTNNNNNGCCCCTGATGAGTTTTGATATTGCCAAATACCCGACCCTGGCA
ipi MAGE	TTCACTCTTCAATTATCTATAATGATGAGTGATCAGAATTACNNNNNAGAAATTATGCAAACGGAACACGTCATTTTATTGAATGCACAG
dxs seqF	ACCAGCAACTTGGTAAAAGTACC
dxs seqR	CGATTTTGTCGCGGCG
ipi seqF	CTCTCTATTCCTGTCATTTCTGACTG
ipi seqR	CAGGAGGCGTAATTTCCACG

Underlined sequences indicate restriction sites.

pINZ-1 was constructed by inserting a fragment containing the pSC101-ts origin and a Km resistance gene into the *pvu*I/*Eco*RV site in the pProbe-GFP[tagless] vector [22]. The pSC101-tsorigin and Km resistant gene were amplified from pKD46 and pKD13, respectively, and spliced together by Splicing by Overlap Extension PCR (SOE-PCR). Thereafter, a 733-bp fragment of the *lacZ* gene was amplified using the primer set lacZF1 and lacZR1 and inserted into the *Xho*I/*Aat*II site of the resultant plasmid. To construct pINZ-2, pINZ-1was amplified excluding the pSC101-ts origin using the primers pKD13R and lacZF, and joined with a fragment containing the R6Kγ origin by SOE-PCR. The latter was previously amplified using primers lacZF2 and lacZR2. pINZ-1-LYC plasmid was constructed by cloning the PCR-amplified *Erwinia herbicola crtEBI* from pAC-LYC into the *Eco*RI and *Sal*I sites of the pINZ1 plasmid [23]. pINZ-1-LYC04 plasmid was constructed by cloning the PCR-amplified *Erwinia herbicola crtEBI* and *Hematococcus pluvialis ipi* genes from pAC-LYC04 into the *Eco*RI and *Sal*I sites of the pINZ1 plasmid [23].

### Plasmid Integration and Excision

For successful integration of the pRED (pRED-1 or pRED-2) or pINZ (pINZ-1 or pINZ-2) plasmids into the *E. coli* MG1655 genome, each plasmid was transformed by electroporation and allowed to recover at a non-permissive temperature (42°C) for the pSC101-ts variant plasmids (pRED-1 or pINZ-1). Transformants with the pRED-2 or pINZ-2 plasmids, carrying the R6Kγ origin, were directly plated with the appropriate antibiotic at 37°C. Selected transformants were confirmed by PCR and DNA sequencing. The *lacZ* null mutants generated by the integration of the pINZ plasmids were selected on X-gal/IPTG plates and recombination efficiency was calculated by estimating the fraction of white colonies in the total number of colonies.

To verify the excision of the pRED-1 plasmid from the genome, the EcSIM1 strain was passaged over two generations at different temperatures (30°C or 42°C), with or without antibiotic selection, and subsequently plated on to LB agar plates. Ten colonies were selected at random and plasmid excision was confirmed by PCR amplification.

### MAGE Conditions

To compare the recombination efficiency of EcNR2 with that of EcSIM, MAGE was performed using a 90-nt oligo (lacZ-MAGE) that introduces a nonsense mutation in the *lacZ* gene as described previously [5]. To show an application of the MAGE process in EcSIM strains, two 90-mer oligos with degenerate ribosome binding site (RBS) (NNNNN; N = A, T, G, C), flanked by the homologous regions of *dxs* and *ipi*, were used to enhance translation efficiency as reported previously [5]. Briefly, an overnight culture was diluted 1∶100 in 3 mL of fresh LB and incubated at 30°C to an OD of 0.5. The *λ* Red system was induced by heating at 42°C for 15 min and cells were immediately chilled on ice. Cells (1 mL) at OD_600_ = 0.5 were harvested by centrifugation at 4°C, made electrocompetent, transformed with 0.5 µM of the oligos and recovered in 1 mL of LB pre-warmed at 37°C. Cells were either re-inoculated into fresh LB for a subsequent round of MAGE or allowed for 3 hours of outgrowth at 30°C prior to selecting the desired recombinants.

To measure the diversity produced by MAGE, genetic variants were transformed with the pINZ-1-LYC04 plasmid containing heterologous genes required for the final steps of lycopene production [23]. The transformants were plated on LB, cultivated at 30°C for overnight, and incubated at room temperature for about 5 days to enhance lycopene production.

## Results and Discussion

### Portable Lambda-Red System for MAGE

**Figure 1:**
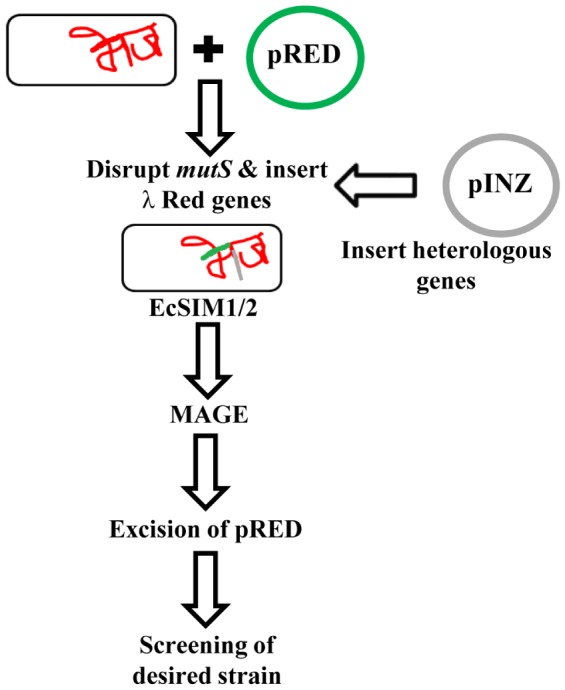
Schematic of the PLRS. The pRED plasmid system delivers λ-Red genes into the *mutS* genomic locus by disruption through a single crossover event between homologues of the partial *mutS* gene. The resultant EcSIM strains can be used for downstream genome engineering process using multiple automated genome engineering (MAGE). Double crossover events at the same locus can result in the excision of the integrated pRED plasmid. Heterologous genes can be inserted into the *E. coli* genome via the pINZ plasmid, which integrates genes of interest into the *lacZ* genomic locus. The resultant EcINZ strains contain foreign genes that can be applied with the pRED system, as illustrated in the text.

**Figure 2:**
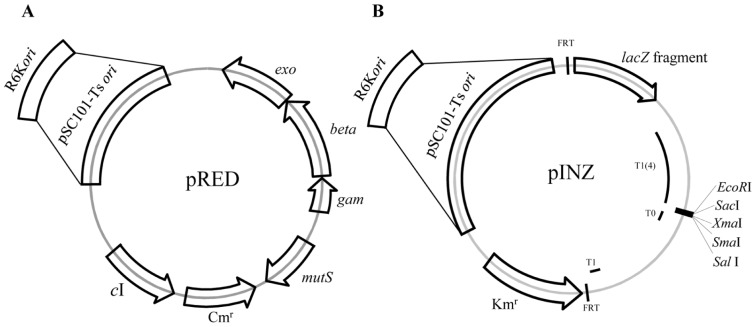
Plasmid maps of the pRED (A) and pINZ (B) systems. FRT, flippase recognition target site; *c*I, λ repressor gene; Km^r^, kanamycin resistance gene; Cm^r^, chloramphenicol resistance gene; *exo*/*beta*/*gam*, λ-Red genes; T1, *E. coli rrnB*1 terminator; T1(4), four tandem *rrnB*1 terminator; T0, λ t0 transcriptional terminator.

The Portable Lambda-Red System (PLRS) includes minimal number of genes (*gam*, *exo*, and *beta*) from the *λ* Red system for recombination and partial *mutS* nucleotide sequences for transient inactivation of the MMR system within a single suicide plasmid (pRED-1 or pRED-2) (Fig. 1). The PLRS also includes a pINZ plasmid system that allows easy integration of heterologous genes into the *lacZ* region of the *E. coli* chromosome (Fig. 2). Recombinants with the suicide plasmid integrated into the chromosome can be selected by exploiting the conditional replicons, pSC101-ts (pRED-1 and pINZ-1) and R6K origin of replication (pRED-2 and pINZ-2) The pSC101-ts origin is active only at low temperatures, whereas the R6K origin is dependent on host cells with the *pir* gene required for its replication (Fig. 2).

**Figure 3:**
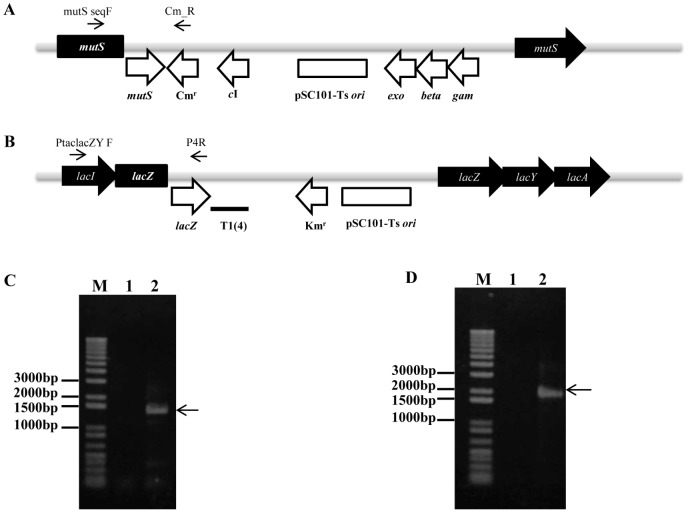
Genomic locus showing integration of pRED1 and pINZ1 into the genome. **A**) pRED plasmid introduces λ Red proteins (Gam, Exo, and Beta) at *mutS* locus genome, generating EcSIM strains. **B**) Heterologous genes can be inserted into the MG1655 genome at the *lacZ* locus through the pINZ plasmid system, generating EcINZ strains. **C**) PCR confirmation of successful inactivation of *mutS* locus by the pRED plasmid system. MG1655 colonies were analyzed for integration of pRED-1 using the following primers: mutSF and Cm_R. Lane M; DNA ladder, lane 1; *E. coli* MG1655 wild type, lane 2; pRED-2 integrated MG1655. **D**) PCR confirmation of successful integration of the *lacZ* locus using the pINZ plasmid system. MG1655 colonies analyzed for integration of pINZ by PCR with primers: Ptac-lacZ and P4R. Lane M; DNA ladder, lane 1; *E. coli* MG1655 wild type, lane 2; pINZ-2-integrated MG1655.

The suicide pRED-1 plasmid was inserted efficiently into the *mutS* region of the chromosome via a single crossover event. The integrants were selected on LB-Cm agar plates at the non-permissive temperature (42°C) (Fig. 1&2) and integration of pRED into the chromosome was confirmed by PCR and sequence analysis (Fig. 2C). The pSC101-ts based selection required several rounds of selection at 42°C for the complete curing of non-integrated plasmids. Therefore, the pRED-1 plasmid was further modified by replacing the pSC101-ts origin with the R6Kγ origin, creating pRED-2, for more efficient selection of plasmid integration in just one round of selection. The R6Kγ origin cannot be replicated in the wild type *E. coli* because of the absence of *pir*, which is derived from the λ *pir* bacteriophage [24]. In the case of pRED-2, none of the transformants were found without chromosomal inactivation of *mutS* by the insertion of the plasmid that has been transformed into MG1655, creating the EcSIM2 strain (Fig. 3).

### Efficiency of Oligo-Mediated Genome Engineering of EcSIM Strains

**Figure 4:**
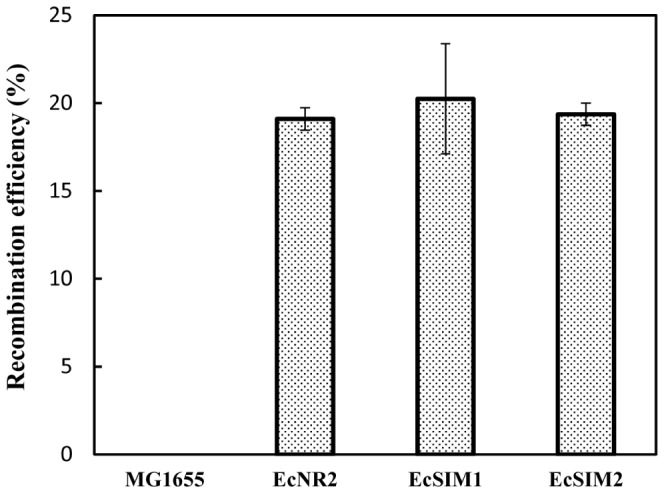
Recombination efficiency of different recombineering strains. 90-nt homologous oligos incorporating a stop codon into the *lacZ* gene were electroporated into *E. coli* cells to analyze the recombination efficiency of the pRED integrated EcSIM strains as compared to EcNR2. Recombinants were selected on X-gal/IPTG plates. The recombination efficiency was calculated by estimating the fraction of white colonies in the total number of colonies.

The full functionality of strains EcSIM1 and EcSIM2 was verified by comparison with well-known recombineering strains like EcNR2. A 90-nt lacZ-MAGE oligo that introduces a nonsense mutation into the *lacZ* gene was used to assess MAGE efficiency in these strains. The recombination efficiency was calculated by counting the percentage of white colonies appearing over two successive rounds of MAGE. Recombination efficiency of EcNR2 was observed to be approximately 20% and is comparable to previously reported results [5]. Recombination efficiency of the newly constructed EcSIM1 or EcSIM2 strain was ≥20%, indicating that the MAGE efficiency is very similar between EcSIM strains containing only the λ Red recombination system and EcNR2, which contain the complete defective λ phage genome (Fig. 4). MAGE with lacZ-MAGE oligo on MG1655 without the expression of λ recombination system did not exhibit any mutations.

### pINZ System

The introduction of heterologous genes into the *E. coli* genome is a common practice in generating novel phenotypes by using genome engineering [25]. Therefore, the pINZ plasmids (pINZ-1 and pINZ-2) were constructed to integrate heterologous genes into the *E. coli lacZ* locus. pINZ plasmids contain a 733-bp partial fragment of *lacZ*, a multiple cloning site, and the pSC101-ts origin (pINZ-1) or R6Kγ origin (pINZ-2) (Fig. 2). As the principle is based on conventional cloning and single crossover mediated integration, the size of heterologous genes may not be a limiting factor for efficient integration. The pINZ-1-LYC carrying three heterologous genes *crtEBI* (4.5 kb) was integrated into the *LacZ* locus, showing integration of a large size of heterologous DNA available for genome engineering. However, this construct was not used for further study because the production levels of lycopene from the genome-integrated pathway were too low to be quantified (data not shown). Additionally, FRT (FLP recognition sites) sites are located upstream of the *lacZ* homologous sequence and downstream of the Km resistance gene for efficient removal of the selection marker upon integration of the target gene. pINZ-1 or pINZ-2 was transformed into the MG1655 strain and selected at 30°C or 37°C, respectively, on LB-Km plates with X-gal and IPTG to generate the EcINZ1 or EcINZ2 strain, respectively (Fig. 3).

### Excision of Integrated Plasmids

*E. coli* strains with a permanently defective MMR system have 100- to 200-fold higher mutation rates than those of the wild type strains [26]. The standard recombineering strain EcNR2 is MMR defective due to the complete deletion of *mutS* gene that encodes a key component of the MMR system. The MMR defective EcNR2 strain can thus lead to the accumulation of undesired mutations. In order to use EcNR2 as production host cells following MAGE, the inactivated MMR system should be rescued. In the case of EcSIM strains, the suicide plasmids (pRED plasmids) can effectively be excised from their chromosomal insertion sites through a second recombination event [27], [28], allowing for only transient inactivation of the MMR system during the genome engineering process.

To address excision efficiency, the pRED-1–integrated EcSIM1 strain was sub-cultured in LB liquid media at a higher non-permissive temperature (42°C) in the absence of antibiotic selection. Excision was confirmed by PCR-amplified target sequence analysis. Plasmid excision occurred at a rate of around 20% after a single subculture (data not shown). This result is comparable to a previously reported result showing an excision efficiency of 10–30% [29], suggesting that the suicide plasmid can be easily eliminated from the host chromosome. The temperature-sensitive plasmid pRED-1 was stably maintained at 30°C with antibiotics, showing no excision event even after three subcultures. Therefore, EcSIM strains appear to be a better alternative to EcNR2, especially for metabolic engineering applications.

### Application of the PLRS for Optimizing the DXP Biosynthesis Pathway

**Figure 5:**
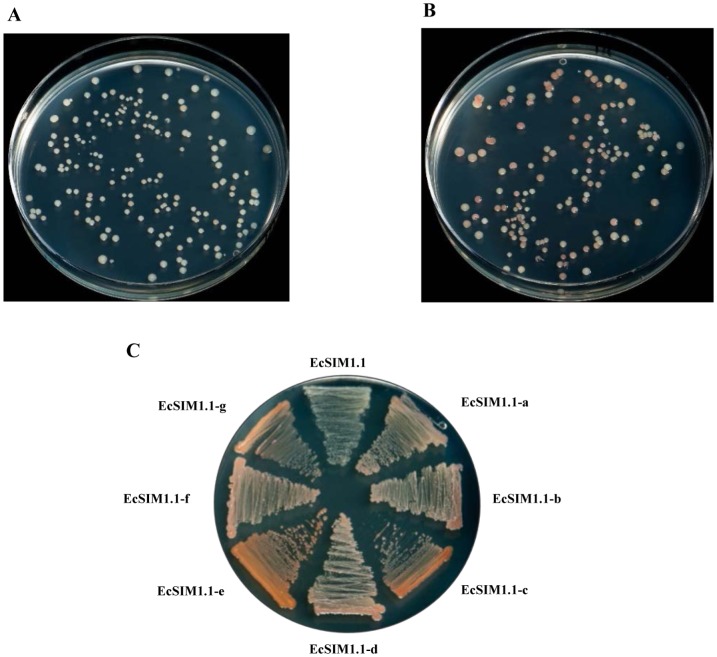
Metabolic pathway engineering of lycopene production by using the PLRS. **A**) Strain EcSIM1.1 was used as a control. **B**) Genomic variant library after 18 cycles of MAGE was grown on LB plate. **C**) Seven representative variants (EcSIM1.1a–g) were compared to the control strain EcSIM1.1 with respect to lycopene production on LB plate.

To demonstrate the efficiency of the PLRS we optimized metabolic flux through the deoxyxylulose-5-phosphate (DXP) biosynthesis pathway. MAGE oligos were designed to change the RBS regions of *dxs* and *ipi*, which encode the rate-limiting enzymes involved in lycopene biosynthesis [5]. After 18 successive cycles of MAGE, the genomic variant library we developed was transformed with the pINZ-1-LYC04 plasmid and was analyzed for lycopene content (Fig. 5). When compared to the control strain EcSIM1.1 (Fig. 5A), differentially pigmented colonies were found in the genomic variant library (Fig. 5B). The efficiency of MAGE was analyzed by PCR amplification and sequencing of the genomic DNA isolated from randomly selected 24 colonies. Among them, 13 strains showed RBS changed in the both genes, one in *dxs*, and four in *ipi*. The representative variants produced intense red pigmentation on the LB plate, indicating high lycopene levels (Fig. 5C), especially in strain EcSIM1.1e. Therefore, our results confirmed that the extensive genomic variant library can be generated effectively in wild-type *E. coli* strains by using the PLRS.

## Conclusions

MAGE, a boon to genome engineering, was previously restricted to use in specialized strains like EcNR2. Additionally, the permanent inactivation of the MMR system of previous MAGE strains limits their use for further screening procedures, such as enrichment-based selection processes, owing to the higher possibility of accumulating secondary mutations. Our PLRS is portable and can be applied to any *E. coli* strain, thus providing opportunities to expand the utility of MAGE. With our PLRS, it is possible to restore a fully functional MMR system upon completion of MAGE, thereby reducing the chance of secondary mutations. Our PLRS is thus suitable for both proficient genome editing and efficient post-selection and screening processes. Here, we expand the modular synthetic biology toolbox with the addition of a completely portable MAGE method applicable to any *E. coli* strain.
